# Optimization of DNA delivery by three classes of hybrid nanoparticle/DNA complexes

**DOI:** 10.1186/1477-3155-8-6

**Published:** 2010-02-24

**Authors:** Qiu Zhong, Dakshina Murthy Devanga Chinta, Sarala Pamujula, Haifan Wang, Xin Yao, Tarun K Mandal, Ronald B Luftig

**Affiliations:** 1 Department of Microbiology Immunology and Parasitology, Louisiana State University Health Sciences Center, New Orleans, Louisiana 70112, USA; 2 College of Pharmacy, Xavier University of Louisiana, New Orleans, Louisiana 70125, USA; 3 Guangdong Food and Drug Vocational College, Guang Zhou, Guangdong 510520, PR China

## Abstract

Plasmid DNA encoding a luciferase reporter gene was complexed with each of six different hybrid nanoparticles (NPs) synthesized from mixtures of poly (D, L-lactide-co-glycolide acid) (PLGA 50:50) and the cationic lipids DOTAP (1, 2-Dioleoyl-3-Trimethyammonium-Propane) or DC-Chol {3β-[N-(N', N'-Dimethylaminoethane)-carbamyl] Cholesterol}. Particles were 100-400 nm in diameter and the resulting complexes had DNA adsorbed on the surface (**out**), encapsulated (**in**), or DNA adsorbed and encapsulated (**both**). A luciferase reporter assay was used to quantify DNA expression in 293 cells for the uptake of six different NP/DNA complexes. Optimal DNA delivery occurred for 10^5 ^cells over a range of 500 ng - 10 μg of NPs containing 20-30 μg DNA per 1 mg of NPs. Uptake of DNA from NP/DNA complexes was found to be 500-600 times as efficient as unbound DNA. Regression analysis was performed and lines were drawn for DNA uptake over a four week interval. NP/DNA complexes with adsorbed NPs (**out**) showed a large initial uptake followed by a steep slope of DNA decline and large angle of declination; lines from uptake of adsorbed and encapsulated NPs (**both**) also exhibited a large initial uptake but was followed by a gradual slope of DNA decline and small angle of declination, indicating longer times of luciferase expression in 293 cells. NPs with encapsulated DNA only (**in**), gave an intermediate activity. The latter two effects were best seen with DOTAP-NPs while the former was best seen with DC-Chol-NPs. These results provide optimal conditions for using different hybrid NP/DNA complexes *in vitro *and in the future, will be tested *in vivo*.

## Introduction

The purpose of this study is to develop a new biodegradable non-viral vector system for the effective transfer of genes to cells and animals. Viral vectors that have been utilized with positive results are adenoviruses with an extremely high transduction efficiency, and adeno-associated viruses (AAV) which are nonpathogenic. Lentivirus (LV) and retrovirus (RV) vectors have also been developed because they can be stably integrated leading to a long lasting genetic transfer. All four approaches are non-toxic and have dominated viral gene therapy efforts in clinical trials and animal models [1-6]. However, after the adverse events which occurred in clinical trials using an RV vector that induced a lymphoproliferative disorder in 2002-2003 [7] due to insertional mutagenesis [8-10], concerns were raised about gene transfer with such a vector. An adenovirus vector also lead to a patient's death in 1999 due to an adverse host immunogenic reaction [11] and AAV vectors still possess an unknown risk with regard to long-term adverse effects [12-14]. Further, viral vectors have their limitations in transfections due to low transgene size; they are expensive to produce and further in many applications they are limited to transient expression [12,13,15,16]. Thus efforts have been directed to develop non-viral gene delivery systems, which include liposome nanoparticles [17,18], the "ballistic" gene gun [19,20], electroporation [21-23] and cationic lipid complexes with DNA [24-28] in vitro and in vivo. However all of these have been beset with issues of cytotoxicity, stability in serum or tissues and like viral vectors, in the duration of gene expression [29,30]. More recent efforts using poly-ethyleneimine (PEI) multilayered materials containing DNA assemblies, as well as blending poly-orthoester (POE) microspheres with branched PEI have been promising as DNA transfection platforms for targeting phagocytic cells [31]. Still, particle size and safety issues with animals remain potential problems with these approaches. Thus, there is a need to establish a biodegradable, stable and long lived nanoparticle vector delivery system. We have established such a system. These are hybrid nanoparticles (NPs) manufactured using the solvent evaporation method [32]. The 100-400 nm particles are derived from a poly (D, L-lactide-*co*-glycolide acid) (PLGA 50:50) base with added cationic lipids (DOTAP or DC-Chol) in organic solution and protamine sulphate in the aqueous solution for enhanced DNA binding ability and increased zeta potential on the NP surface [33]. Using this procedure, molecules for gene therapy (plasmid DNA, antisense oligonucleotide, small interfering RNA) can be adsorbed on the surface or encapsulated into the NPs. An advantage of this method is that the simple evaporation process is performed under mild physicochemical conditions and leads to improved nucleic acid absorption. This method requires dissolving both polymers and lipids in non-aqueous phase and nucleic acid in the aqueous phase.

In previous studies, we have used agarose gel electrophoresis to demonstrate that plasmid DNA can be bound and released from cationic microparticles [34,35]. Here we improve upon these studies by using the luciferase gene as a sensitive marker for DNA activity in transfected cells. Overall, three classes of DNA adsorbed and/or encapsulated hybrid NPs were formulated; they were designated as DNA adsorbed (**out**), DNA encapsulated (**in**), and DNA adsorbed/encapsulated (**both**) NPs. The release profile of DNA from PLGA/DOTAP or PLGA/DC-Chol adsorbed NPs (**out**) after transfection with 293 cells exhibited a large initial uptake followed by a rapid DNA decline over a four week period. This was based on the measurement of luciferase activity in 293 cells at 3-4 day intervals. The encapsulated (**in**) and adsorbed/encapsulated (**both**) NPs also showed an initial uptake, but was followed by a period of gradual DNA degradation seen by a sustained and a slow release of encapsulated DNA in the 239 cells. Hybrid NPs as constituted should provide an effective alternative to viral gene therapy. Recent applications of similar PLGA/DOTAP NP technology, using an asialofetuin ligand complexed with the therapeutic gene IL-12 look promising in this regard [36].

## Methods

### Materials

1, 2-Dioleoyl-3-Trimethylammonium-Propane (Chloride Salt) (DOTAP) and 3β-[N-(N', N'-Dimethylaminoethane)-carbamoyl] cholesterol hydrochloride (DC-Chol) were purchased from Avanti Polar Lipid (Alabaster, AL). The copolymer poly (D, L-lactic-co-glycolic acid), PLGA 50:50 (RG 502; inherent viscosity 0.2 dL/g) was obtained from Boehringer Ingelheim (Germany) and Protamine Sulphate (PS) was from Sigma (St. Louis, MO). The reporter plasmid DNA pGL4.75 (pLuc) containing the *Renilla *luciferase gene and Luciferase assay kit were purchased from Promega (Madison, MI). Lipofectamine™ 2000 (Lip2000) was obtained from Invitrogen (Carisbad, CA).

### Cell Culture

Adherent 293 and PC-3 human prostate tumor cells were from ATCC (Manassas, VA) and maintained at 37°C in 5% CO_2 _in Dulbecco's modified Eagle's medium (DMEM) supplemented with 10% (v/v) heat-inactivated fetal bovine serum (FBS) and 1% (v/v) penicillin (5,000 U/ml), and streptomycin (5,000 μg/ml) from Invitrogen (Carisbad, CA). The adherent LNcap human prostate tumor cells and the non-adherent suspension MOLT-4 human T lymphoblast cell line from ATCC were maintained in RPMI-1640 Medium supplemented with serum and antibiotics, as above. All cells were passaged 1:4 twice a week.

### Preparation of PLGA/DOTAP or PLGA/DC-Chol Hybrid Nanoparticles

**Figure 1:**
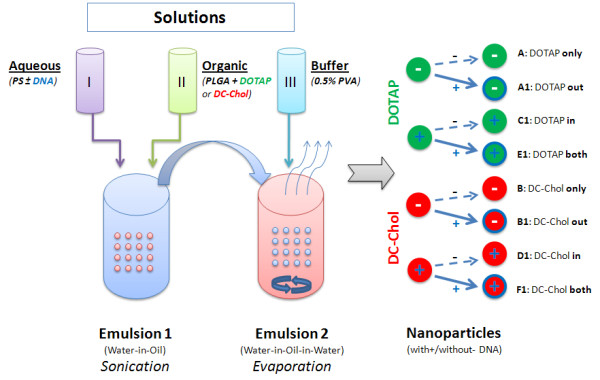
**Nanoparticle preparation: Emulsion 1 (W/O) was obtained after an aqueous buffer containing Protamine Sulphate (PS) +/- DNA (blue) (solution I) was mixed with an organic buffer of PLGA with cationic lipids DOTAP (green) or DC-Chol (red) (solution II) and sonicated**. Then another aqueous buffer containing PVA (solution III) was added to form Emulsion 2 (W/O/W). The mixture was briefly sonicated and NPs were formed by solvent evaporation. For DNA encapsulated NPs (**in **and **both**), pLuc DNA was added to solution I. For DNA adsorbed NPs (**out **or **both**), pLuc DNA was added to the NPs as described in the methods. The nanoparticles are designated as: green for PLGA/DOTAP, red for PLGA/DC-Chol and a blue plus inside the circle for encapsulated DNA. Blue on the outer circle designates adsorbed DNA.

PLGA is an FDA approved biodegradable polymer [37]. The PLGA-Lipid hybrid NPs with and/or without DNA were formulated by using a double emulsion (W/O/W) - solvent evaporation method (Figure 1). Briefly, the first or aqueous solution (Solution I) Tris-EDTA buffer (pH 8.0) was mixed with PS plus DNA for future inside (**in**) or **both **NPs or PS minus DNA for future outside (**out**) NPs. After adding the organic solution (Solution II) of 40% (w/v) PLGA with cationic lipid (DOTAP or DC-Chol), the water-in-oil (W/O) emulsion was sonicated at output 4 (50 W) for 30 seconds (ultrasonic probe, Sonic & Materials Inc., Danbury, CT, USA). Then it was transferred to an aqueous buffer (Solution III) containing 0.5% PVA and sonicated for 15 min at 30% amplitude. The resultant water-in-oil-in-water (W/O/W) emulsion was stirred for 18 hrs at room temperature with a magnetic stirrer until all of the organic solvent had evaporated. The NPs were collected by centrifugation at 35,000 rpm for 20 minutes at 10°C (Beckman Coulter-Optima L-100 XP Ultra Centrifuge, Fullerton, CA, USA), washed four times with TE buffer, and freeze dried at -20°C for 48 hrs. The pLuc DNA was adsorbed to NPs for preparation of (**out **or **both**) NPs by overnight incubation at 4°C using the concentrations shown in Tables 1 and 2.

**Table 1:** Composition of nanoparticles complexed with DNA on the surface (out)

Formulation	Cationic Particles		DNA	Protamine Sulphate
A1 (out)	DOTAP (A)	10 mg	250 μg	150 μg
B1 (out)	DC-Chol (B)	10 mg	250 μg	150 μg

A: PLGA/DOTAP-NPs B: PLGA/DC-Chol-NPs

**Table 2:** Composition of NPs with DNA encapsulated (in) or adsorbed and encapsulated (both)

Formulation		Solutions					NP Surface Modifications (out)	
				
		I		II		III		
				
		PS	DNA	PLGA	Lipid	Buffer	DNA	PS
C	(in)	450 μg	750 μg	30 mg	6.5 mg (DO)	6 ml	------	------
D1	(in)	450 μg	750 μg	30 mg	6.5 mg (DC)	6 ml	------	------
E1	(both)	112 μg	187 μg	15 mg	3.25 mg (DO)	3 ml	187 μg	112 μg
F1	(both)	112 μg	187 μg	15 mg	3.25 mg (DC)	3 ml	187 μg	112 μg

PS: Protamine Sulphate DO: DOTAP DC: DC-Chol Buffer: 0.5% of PVA in Buffer

### Particle Size, Zeta potential and Morphology of Nanoparticles

Particle size distribution and Zeta Potential were determined by a Delsa™ Nano C Zeta Potential and Submicron Particle Size Analyzer (Beckman Coulter Inc., Fullerton, CA, USA), using photon correlation spectroscopy (PCS). In this technique, the particle sizes are determined by measuring the rate of fluctuations in laser (30 mW dual laser) light intensity scattered by particles as they diffuse through a fluid. The NPs (0.5 mg) dispersed in deionized water were added to a cell holder and counting was performed (70 accumulation times). Each experiment was performed in triplicate. The particle zeta potentials are determined by measuring the electrophoretic movement of charged particles under an applied electric field. The Delsa instrument used a zeta potential module equipped with a 35 mW two laser diode (658 nm). Scattered light was detected at a 90 angle and a temperature of 25°C. About 1.6 ml of a suspension of charged particles in water was used for the measurements. Zeta potential values (Tables 3 and 4) were calculated from measured velocities using the Smoluchowski equation.

**Table 3:** Physical properties of PLGA cationic particles

Formulation		Particle Size (nm)			Zeta Potential (mv)
			
		d (0.1)	d (0.5)	d (0.9)	
A	PLGA/DOTAP	95	218	425	52.64 ± 1.17
B	PLGA/DC-Chol	86	210	523	41.67 ± 2.55

The mean size and distribution for different NPs are indicated; d(0.1), d(0.5), d(0.9) means that less than 10%, 50%, 90% of the NPs respectively, are distributed around the particle sizes indicated

The shape and surface morphology (smooth versus porous structure) of the nanoparticles were investigated using a scanning electron microscope (SEM) (S-4800N, Tokyo, Japan). Nanoparticles suspended in deionized water were freeze-dried. The dried nanoparticles were mounted on metal stubs with double sided tape and coated with a thin gold layer using an ion coater (K550X, EMITECH, Kent, UK).

### Quality Control for DNA Location on Nanoparticles

**Figure 2:**
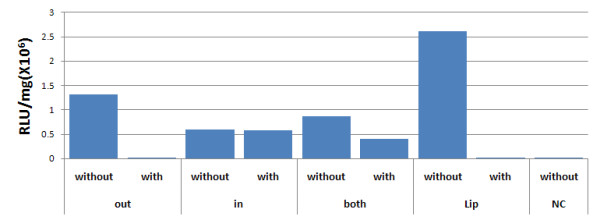
**Quality control for pLuc DNA adsorbed to either surface NPs (out and both) or encapsulated NPs (in and both)**. The NP/DNA complexes were treated with or without DNase I and delivered to 293 cells for 48 hours. Lipofectamine 2000 with pLuc DNA was a positive control (Lip) and untreated 293 cells was the negative control (NC). The assay measures luciferase activity.

We used measurement of luciferase activity for transgene expression, as the most sensitive assay to assign DNA location (**out, in **or **both**) on the different NP/DNA complexes. The six NPs were each suspended in water, treated with DNase I (Fermentas, Glen Burnie, MD) at 37°C for 30 min, washed and delivered to 293 cells. Specifically, 16 μg NPs (with or without DNase I treatment) were added to 10^5 ^cells in 48 well plates for 48 hours and luciferase activity was measured as seen in Figure 2. We had previously tried unsuccessfully, to measure residual DNA by location on the NP/DNA complexes, using DNA concentration (OD at 260 nm) or agarose gel electrophoresis before and after DNase I digestion.

### Evaluation of NP/DNA Complex Uptake in vitro by Cells

For dose responses assays, 293 cells were seeded onto 48 well plates at a density of 10^5 ^cells per well in 1 ml DMEM (Invitrogen, Carisbad, CA) containing 10% FBS. Incubation of cells was for 24 hr at 37°C in a 5% CO_2 _incubator. Each of the six different NPs in 50 μl PBS and containing pLuc DNA was added at concentrations of 164 ng to 100 μg (in 2 to 2.5 fold-stepwise intervals) to separate wells. After 48 hrs incubation, luciferase was assayed using a kit from Promega. DNA with Lip2000 was the positive control (PC) and DNA only was the negative control (NC).

Regression analysis and determination of the declination angles for DNA uptake of NPs by 293 cells was performed using the trend line program from a Microsoft Excel 2007 software statistical package. Cells were passaged at 10^5 ^cells per ml in a T25 flask containing 5 ml DMEM with 10% FBS. After 24 hr, each of the six NPs containing pLuc DNA was added at 40 μg and culturing was maintained for up to 4 weeks. At 3 or 4 day intervals, cell density was adjusted to 10^5 ^cells per ml by adding fresh medium. DNA activity was measured by the luciferase assay.

## Results and Discussion

### Characterization of hybrid nanoparticle/DNA complexes

**Figure 3:**
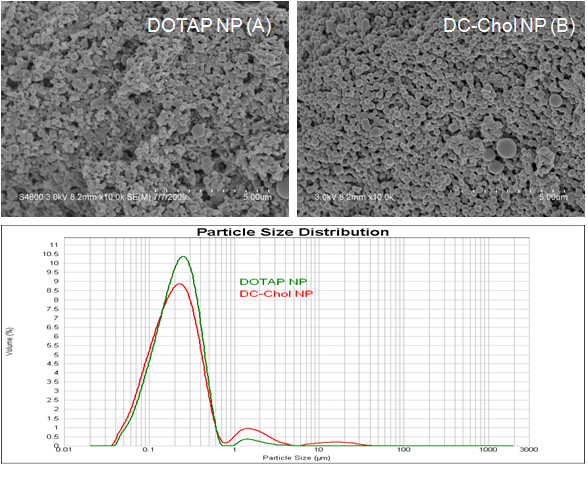
**SEM photomicrograph of PLGA/DOTAP and PLGA/DC-Chol nanoparticles (top)**. The corresponding particle size distribution for PLGA/DOTAP nanoparticles (green) and PLGA/DC-Chol nanoparticles (red) is on the bottom.

PLGA based NPs prepared by the solvent evaporation method (Figure 1), with either DOTAP or DC-Chol showed a similar particle size distribution (Figure 3). From the representative size distribution diagrams, it can be seen that in both formulations 70% of particles were in the range of 100-400 nm. NPs formulated, either with DOTAP or DC-Chol, exhibit a uniform spherical shape with smooth surface as seen by scanning electron microscopy. The particle size distributions and zeta potentials are described in Table 3. Initially, PLGA NPs with PVA, a most commonly used surfactant or stabilizer, have a negative surface charge because of physical entrapment of liquid within the surface layer of the polymer [38]. In our formulations, after addition of cationic lipids (DOTAP and DC-Chol) an overall positive charge is imparted to the NP surface. The PLGA/DOTAP and PLGA/DC-Chol NPs also were complexed with luciferase gene plasmid DNA pLuc (pGL4.75), at the concentrations described (Table 1, 2). Although the zeta potential is varied in all formulations, it is still positive in all cases. The lower positive zeta potentials of adsorbed NPs (**out **and **both**) may possibly be due to the nullifying effects of negative charge on DNA versus the positive charge of cationic lipid on the surface of these NPs, compared to encapsulated NPs (**in**) (Table 4). Previous studies with such cationic lipid/DNA NP complexes have shown that they are stable [34] and efficiently taken up by tissue culture cells [35,39]. In this study we have focused on delivery of such NPs to 293 and other cells.

**Table 4:** Zeta potential of nanoparticle DNA complexes

Formulation			Zeta Potential (mv)
A1	DOTAP	(out)	06.86 ± 0.72
B1	DC-Chol	(out)	05.83 ± 0.24
C1	DOTAP	(in)	31.95 ± 0.99
D1	DC-Chol	(in)	14.84 ± 0.11
E1	DOTAP	(both)	16.40 ± 0.27
F1	DC-Chol	(both)	06.46 ± 0.07

DOTAP: PLGA/DOTAP DC-Chol: PLGA/DC-Chol

### Optimization of NP DNA binding conditions

We determined the optimal conditions for binding the maximal amount of DNA to the PLGA hybrid NPs. The two types, DOTAP (A) or DC-Chol (B) hybrid NPs, were complexed with luciferase gene plasmid DNA at a w/w ratio of 10/1 and held at 4°C, room temperature (22°C) or 50°C for 1, 2, 3, 4 hours, as well as overnight. Both types gave similar results, so we will describe specific findings for DOTAP/DNA NPs (**out**). After 3 hours at 4°C or 22°C these NPs have a similar, high level of DNA binding activity relative to those held at 50°C. 100 μg of such NP/DNA complexes formed at 4°C or room temperature were then transferred for uptake to 10^5 ^293 cells in 1 ml and incubated for 1 day. About a 23% increase in DNA binding was observed at 4°C. The maximal amount of DNA that could tightly bind to the NPs at 4°C was then determined. For this, NP/DNA (w/w) ratios of 10/1 to 50/1 were incubated overnight at 4°C. Then the NPs were pelleted and the supernatant was collected. DNA measurements were made both for the NP/DNA complexes and free DNA using 1 mg of NP complexed with 100 μg, 50 μg, 40 μg and 20 μg of DNA. The amount of free DNA was highest at the 10/1 ratio and lowest at the 50/1 ratio; however all levels showed that ≥ 95% of DNA was bound to the NP. Based on these findings, our experiments utilized NPs at a ratio of 20-30 μg DNA/1 mg NP, in order to avoid competition with free DNA.

### Localization of DNA in the nanoparticles/DNA complexes

The six NP/DNA complexes were suspended in water at 10 mg/ml. In order to verify DNA location on the outside or inside of the NP complexes respectively, we used the following approach to determine sensitivity to DNase I. NP/DNA complexes were treated with DNase I and delivered to 293 cells. Expression of residual DNA was assigned by measuring luciferase activity after 48 hours. We note in Figure 2 that those NP/DNA complexes where DNA was adsorbed on outer surfaces (**out **and **both**) were able to be cleaved by DNase I. Thus no expression was detected for **out**, but about 50% expression was detected for **both**. As expected, no difference was seen for NPs with encapsulated DNA (**in**) (Figure 2).

### Optimization of NP/DNA complex delivery conditions to 293 cells

**Figure 4:**
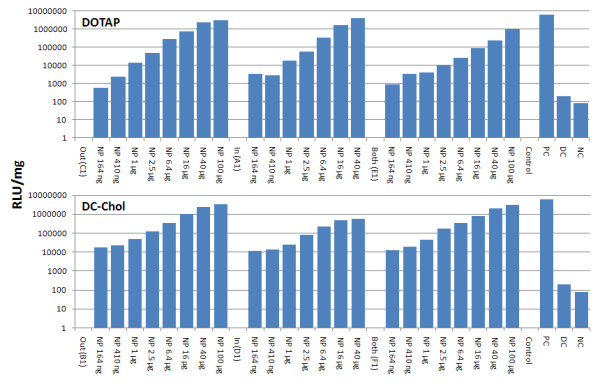
**Dose/response bar graphs showing efficiency of DNA delivery to 293 cells after 48 hours incubation for three classes of NPs made from two type of cationic lipid; DOTAP (top) and DC-Chol (bottom)**. NP/DNA complexes were added at concentrations from 164 ng to 100 μg in 2.5 fold-stepwise intervals. Positive control (PC) is Lipofectamine 2000 with 100 ng DNA; DNA control (DC) uses 10 μg DNA alone; Negative control (NC) is 293 cells only and no particles, lipofectamine or DNA.

**Figure 5:**
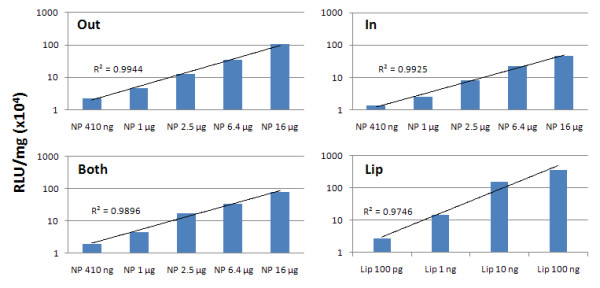
**Dose/response bars and lines showing transfection efficiency**. Luciferase activity was measured (blue bars) and the corresponding straight lines generated (black lines). DOTAP NPs (25 μg DNA/mg NPs) were added at amounts of 410 ng to 16 μg NPs to 10^5 ^cells/ml (293 cells) for 48 hours. Top shows **Out **and **In **NP/DNA complexes. Bottom shows **Both **NP/DNA and Lip2000 (Lip) complexes; Lip/DNA complexes were added at 100 pg to 100 ng DNA.

We compared the efficiency of DNA delivery to 293 cells by the six NP/DNA complexes vs. a Lip2000/DNA mixture. Lipofectamine 2000 is a cationic lipid widely used to transfect plasmid and other DNA into a variety of mammalian cells. Invitrogen reports [40] that 293 cells transfected with pCMV-β gal DNA exhibited a high transfection efficiency (99%) and 100% cell viability at 24 hours post transfection. PLGA/DOTAP or PLGA/DC-Chol NPs with the composition of pLuc DNA seen in Tables 1 and 2 were formulated as in Figure 1, and all six were used at a concentration of 25 μg DNA/1 mg NP. NPs were added to 10^5 ^cells at 2 to 2.5 fold increasing concentrations starting at 164 ng and going to 100 μg for 2 days (Figure 4). Based on the R^2 ^value of the straight line seen in Figure 5 for the three DOTAP NP/DNA complexes, the transfection efficiency achieved is high and similar to that for Lip2000/DNA complexes.

Although Lipofectamine 2000 appears effective at lower concentrations of plasmid DNA (100 pg to 100 ng), it has the disadvantage of toxicity, as noted in the introduction and thus would have limited applicability *in vivo*. Specifically, high cytotoxicity in renal and arterial tissue-based studies [41,42], as well as in animal applications [43,44] have been reported. Hybrid NPs in contrast, are safe in cell and animal studies [41,45]. Further, from Figures 4 and 5 we note that NPs are best used at concentrations of 16-40 μg NPs/ml with 293 cells; NP levels ≥ 100 μg/ml are cytotoxic (data not shown). The DNA binding experiment seen in Figure 5 was repeated with DC-Chol NPs and gave a similar result. The relative transfection efficiency of pLuc DNA calculated from these experiments show that DOTAP or DC-Chol NPs are nearly as efficient as Lip2000 in delivering DNA to 293 cells; however, when compared to free DNA, NPs have a 500-600 fold higher transmission efficiency. In conclusion, we find that after 2 days of NP/DNA complex delivery to 293 cells (Figure 4), "**Out**" NPs shows a higher luciferase expression than NPs with only inside DNA (**in**) and luciferase expression is intermediate for "**Both**" NPs. This suggests that outside DNA exhibits an initial high expression due to rapid release of bound DNA. On the other hand, DNA encapsulated NPs (**in**) are slower to release DNA and are probably affected by biodegradation of the NPs within cells.

### Study of gene delivery with hybrid nanoparticle/DNA complexes using other cell lines

The optimal condition for DNA gene delivery to 293 cells was shown in Figures 4 and 5, and we found that all six NP/DNA complexes showed a high efficiency of gene transfection. We also were interested in checking transfection with other cell lines and found that two adherent prostate cell lines (PC-3, LNcap) gave the same high efficiency for the six different hybrid NP/DNA complexes, again compared to Lip2000 (data not shown). Interestingly, when non-adherent MOLT-4 cells were used, only a high transfection efficiency was found with the NP/DNA complexes and not Lip2000 (data not shown).

### Degradation of NP/DNA complexes delivered to 293 cells

**Figure 6:**
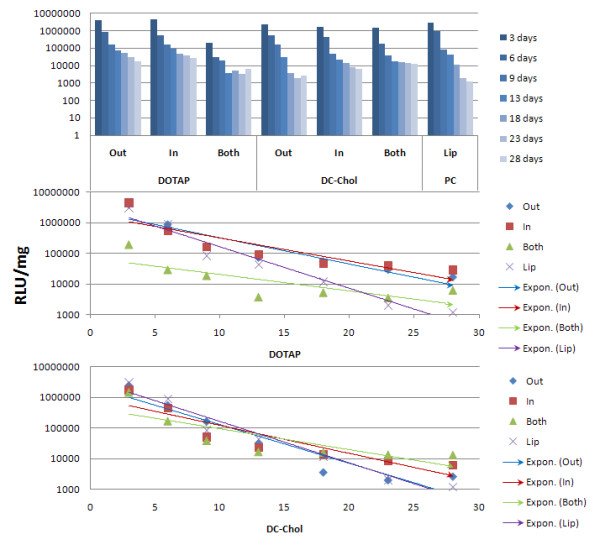
**Degradation analysis for DNA delivery to 293 cells by six different nanoparticle/DNA complexes over a four week period**. Two NP/cationic lipid mixtures (PLGA/DOTAP and PLGA/DC-Chol) and three classes of NP/DNA complexes (**out**, **in **and **both**) were used. Lip (Lip2000/DNA mixture) was a positive control. Top columns show luciferase activity at 3 or 4 day intervals for 4 weeks. Middle graph is (DOTAP) and bottom graph (DC-Chol) NPs. Regression analysis gave straight lines (blue for **out**, red for **in **and green for **both**) for nanoparticles and Lip (purple).

For these experiments, we freshly prepared the six NP/DNA complexes, using a NP/DNA (w/w) ratio of 40/1 (Figure 1). Such complexes bound DNA at a level of 96% to 99%. They were added to 293 cells for 3 days and incubated at 37°C for about 4 weeks. Cell passages were done at 3 to 4 day intervals. Samples were removed at these times and the level of luciferase DNA was measured. The results are shown in Figure 6 with a positive control using Lipofectamine (Lip). The top figure presents the data in a graph format, while the middle and bottom provide the data as straight lines. These results represent the release profile of DNA from the NP/DNA complexes within 293 cells, over time. Regression analysis was performed and lines were drawn of the data points taken for the 4 week period. DC-Chol NPs containing externally bound DNA (**out**) (bottom graph) exhibited a large initial uptake followed by a steep decay of pLuc DNA, similar to Lipofectamine. However with DOTAP (middle graph), externally bound DNA NPs (**out**) exhibited a diminished slope of DNA decay relative to Lipofectamine. DOTAP NPs (middle graph) and DC-Chol NPs (bottom graph) with bound and encapsulated DNA (**both**) also led to a large initial uptake, but it was followed by sustained DNA release over a longer time. This is correlated with a lower angle of declination of the regression line than Lip (average angle of 23.8° for DOTAP and 29.3° for DC-Chol) (Table 5). NPs with only encapsulated DNA (**in**) showed an intermediate level of DNA degradation. Since all assays started with the same number of cells, this different decline in luciferase activity with different NPs is not likely to be a cell dilution problem. In summary, the "**Lip**" and "**Out**" NP complexes have similar profiles (steep slope) because both have outside bound DNA and the expression assay in 293 cells reflects the rapid release of such bound DNA. On the other hand, "**In**" and "**Both**" have longer retention profiles, indicating that this expression assay is affected by biodegradation in time, of encapsulated NP/DNA complexes within cells. However, our results show that the "**Both**" NP/DNA complexes, which have DNA both outside and inside show a higher level of luciferase activity after four weeks than the "**In**" NP/DNA complexes. This may be because the former NPs with DNA on the outside can stabilize the surface charge and allow for a longer retention time within 293 cells. These findings are important for the future design of vaccines using NP/DNA complexes. Thus, when an initial strong gene delivery response over a short time is required, as in "priming" for an antibody in animals, it appears that NP complexes with adsorbed DNA (**out**) are best used. However, for a response where one wants a longer time of gene delivery, as in a "booster" inoculation, the adsorbed/encapsulated DNA complexes (**both**) are best used. It should be noted with NPs that there is always the potential for an inflammatory response as with gene delivery systems, but in both cases this is usually dependent on immune response to the transgene product.

**Table 5:** Angle of regression line declination* over a four week period for six nanoparticle preparations

Experiment	DOTAP			DC-Chol		
	out	in	both	out	in	both
#1	35.5°	32.3°	25.3°	46.8°	35.9°	29.5°
#2	30.1°	28.1°	17.4°	39.2°	32.0°	23.7°
#3	42.3°	36.5°	28.7°	54.5°	36.5°	34.6°

Average	36.0°	32.3°	23.8°	46.8°	34.8°	29.3°

*Angle is in degrees and reflects pLuc DNA degradation over time in 293 cells

## Conclusion

Nanoparticles provide a better vector than DNA alone for luciferase gene delivery (500-600 times more efficient). A dose response curve for gene delivery of six different NP/DNA complexes to 293 cells has been generated; optimal delivery conditions occur for 10^5 ^cells over a range of 500 ng-10 μg of NPs containing 20-30 μg DNA per 1 mg of NPs. NPs with externally bound DNA (**out**) led to a steep slope on lines drawn from regression analysis, while NPs with both adsorbed and encapsulated DNA (**both**) exhibited a longer retention time. This offers the potential of using hybrid NPs with adsorbed DNA (**out**) for "priming" in animal immunization studies, while DNA adsorbed/encapsulated NPs (**both**) are optimal for "booster" immunization.

## Competing interests

The authors declare that they have no competing interests.

## Authors' contributions

QZ carried out design and performed study, data analysis and drafting of the manuscript. TKM directed, while DMDC and SP carried out NP formulation and characterization such as particle size, zeta potential and morphology of nanoparticles. HW consulted and participated in the design of the study. XY carried out the Luciferase assay in evaluation of NPs and prepared cells. RBL was involved with the design, coordination, data analysis and drafting of the manuscript through its many revisions. All authors read and approved the final manuscript.
